# Supplementary material for: Structure, interaction and nervous connectivity of beta cell primary cilia
Source: Nat Commun. 2024 Oct 24;15:9168. doi: 10.1038/s41467-024-53348-5 (PMC11502866; doi:10.1038/s41467-024-53348-5)
Supplement: Supplementary file 1 — Supplementary Information [file 41467_2024_53348_MOESM1_ESM.pdf]

Supplementary Figures

Structure, interaction and nervous connectivity of beta cell primary cilia

Andreas Müller[1,2,3,#,*], Nikolai Klena[4,#], Song Pang[5,6], Leticia Elizabeth Galicia Garcia[1,2,3,7], Oleksandra Topcheva[1,2,3], Solange Aurrecoechea Duran[1,2,3], Davud Sulaymankhil[1,2,3,8], Monika Seliskar[1,2,3], Hassan Mziaut[1,2,3], Eyke Schöniger[1,2,3], Daniela Friedland[1,2,3], Nicole Kipke[1,2,3], Susanne Kretschmar[9], Carla Münster[1,2,3], Jürgen Weitz[10], Marius Distler[10], Thomas Kurth[9], Deborah Schmidt[11], Harald F. Hess[5], C. Shan Xu[5,12], Gaia Pigino[4,#,*], and Michele Solimena[1,2,3,7,#,*]

[1]Molecular Diabetology, University Hospital and Faculty of Medicine Carl Gustav Carus, TU Dresden, Dresden, Germany

[2]Paul Langerhans Institute Dresden (PLID) of Helmholtz Munich at the University Hospital Carl Gustav Carus and Faculty of Medicine of the TU Dresden, Dresden, Germany

[3]German Center for Diabetes Research (DZD e.V.), Neuherberg, Germany

[4]Human Technopole (HT), Milan, Italy

[5]Janelia Research Campus, Howard Hughes Medical Institute, Ashburn, VA, USA

[6]Current address: Yale School of Medicine, New Haven, CT, USA

[7]DFG Cluster of Excellence "Physics of Life", TU Dresden, Dresden, Germany

[8]Current address: Department of Chemical Engineering, Cooper Union, NYC, NY, USA

[9]Center for Molecular and Cellular Bioengineering (CMCB), Technology Platform, Core Facility Electron Microscopy and Histology, TU Dresden, Dresden, Germany

[10]Department of Visceral, Thoracic and Vascular Surgery, University Hospital Carl Gustav Carus, Medical Faculty, TU Dresden, Dresden, Germany

[11]HELMHOLTZ IMAGING, Max Delbrück Center for Molecular Medicine (MDC) in the Helmholtz Association, Berlin, Germany

[12]Current address: Department of Cellular & Molecular Physiology, Yale School of Medicine, New Haven, CT, USA

# Authors contributed equally

* Corresponding authors: andreas.mueller1@tu-dresden.de, gaia.pigino@fht.org, michele.solimena@uniklinikum-dresden.de

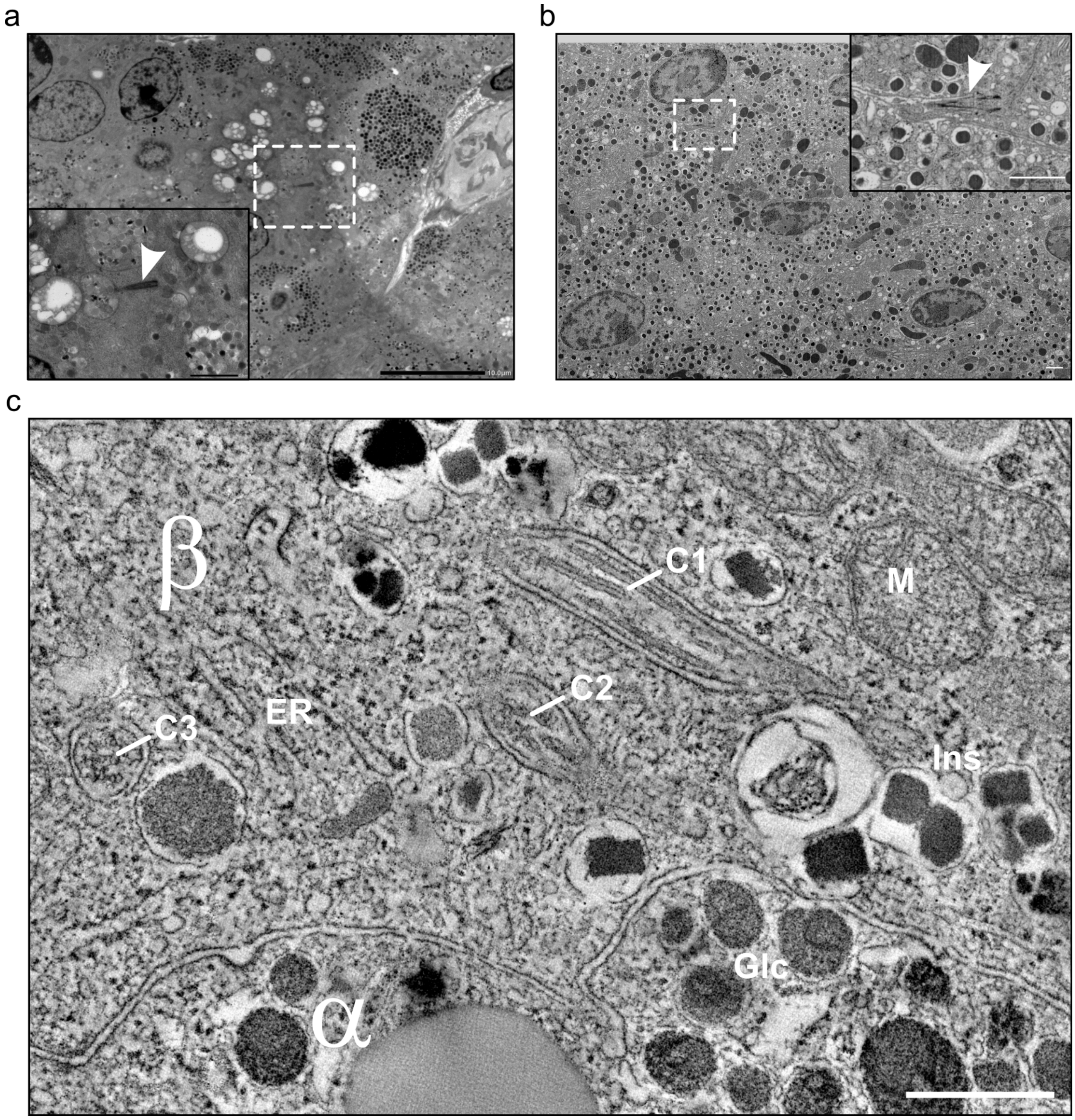

Supplementary Figure 1: Identification of primary cilia in ssET and FIB-SEM

a) TEM overview of a 300 nm thick section of human pancreas. Inset shows a primary cilium identified by the axoneme structure. Scale bars: 10 and 1 µm. b) FIB-SEM slice of mouse pancreatic islets. The inset shows a primary cilium. Scale bars: 1 µm. c) Single slice of a tomogram with a beta cell (β) with insulin secretory granules (Ins), mitochondria (M), endoplasmic reticulum (ER) and cilia (C1-3). The neighboring alpha cell (α) with glucagon granules (Glc). Scale bar: 1 µm.

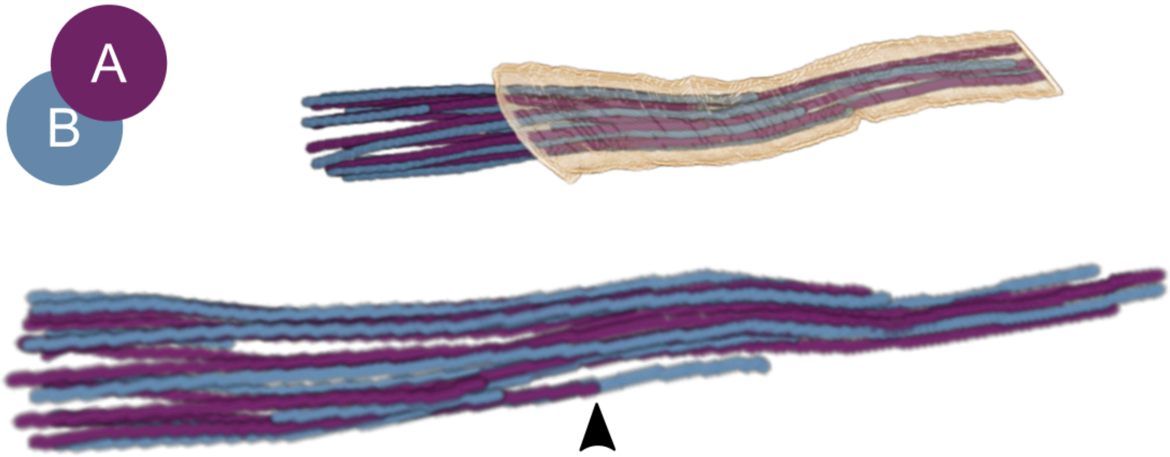

Supplementary Figure 2: Reconstruction of an incomplete human beta cell primary cilium with longer B-tubules
3D rendering of A-(purple) and B-(blue) tubules with ciliary membrane (light orange). Below is a magnified rotated rendering with an arrowhead pointing to the earlier determination of the A-tubule of the specific microtubule doublet.

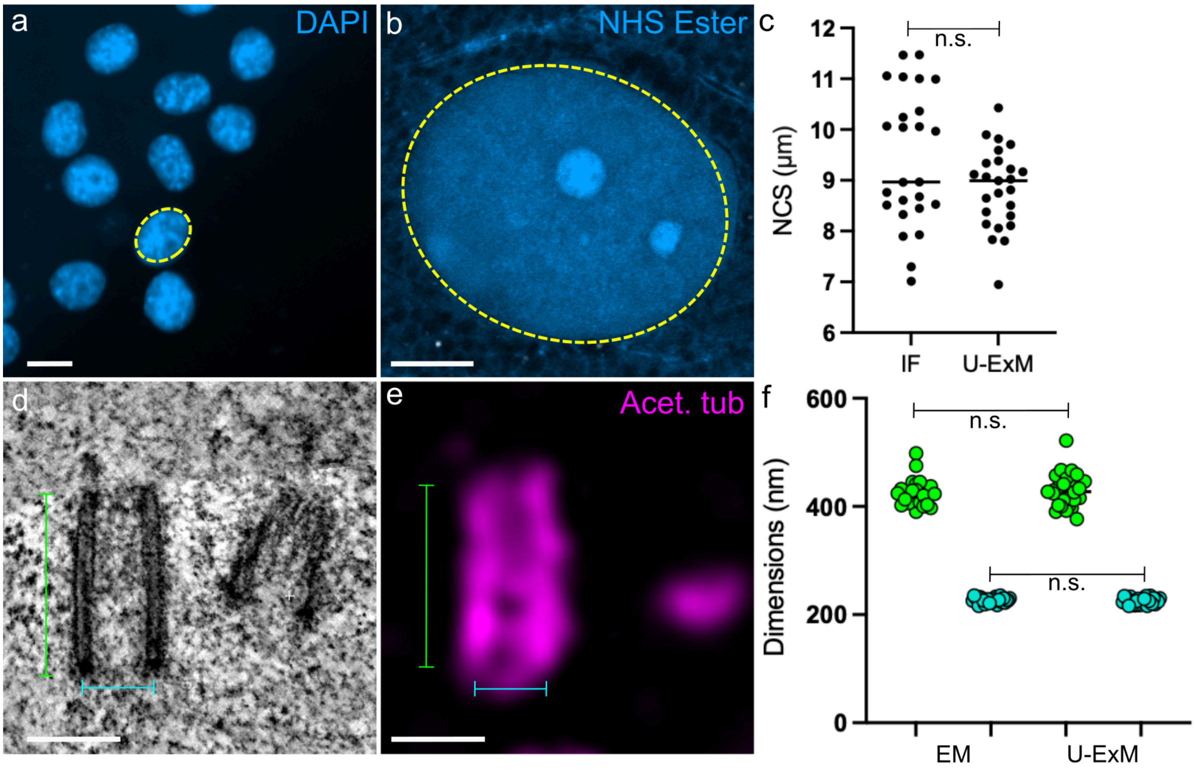

Supplementary Figure 3: Validation of U-ExM isotropy in expanded pancreas tissue.

a) Single slice of a mouse islet stained for DAPI (blue). Scale bar: 10 µm. b) Single slice of an expanded mouse beta cell stained with NHS ester (blue). Scale bar: 2.5 µm. Dotted yellow lines in (a, b) demarcate the representative nuclei area measurements made in (c). c) Nucleus cross-section area (NCS) = Square root of area / measured gel expansion factor) in unexpanded beta cells (IF) vs. expanded beta cells (U-ExM). Mean NCS: unexpanded beta cells, 9.428 µm +/- 1.3, expanded beta cells, 8.842 µm +/- 0.7871. Error reported in standard deviation. Statistical significance measured by unpaired t-test. $p = 0.637$. d) Representative FIB-SEM micrograph of daughter centriole. Scale bar: 300 nm. e) Representative U-ExM micrograph of daughter centriole, scale bar: 300 nm. Green lines and cyan lines in (d, e) demarcate the length and width measurements, respectively, displayed in (f). Length and width measurements of the daughter centriole by FIB-SEM (EM) vs. expansion (U-ExM). Mean dimensions: EM length 425.2 nm +/- 24.15, U-ExM length, 428.9 nm +/- 31.59, EM width, 226.5 nm +/- 5.375, U-ExM width, 225.5 nm +/- 6.349. Error reported in standard deviation. Statistical significance measured by unpaired t-test. $p = .6379$, EM length - U-ExM length, $p = .5611$ EM width vs. U-ExM width. $n = 25$ daughter centrioles per condition.

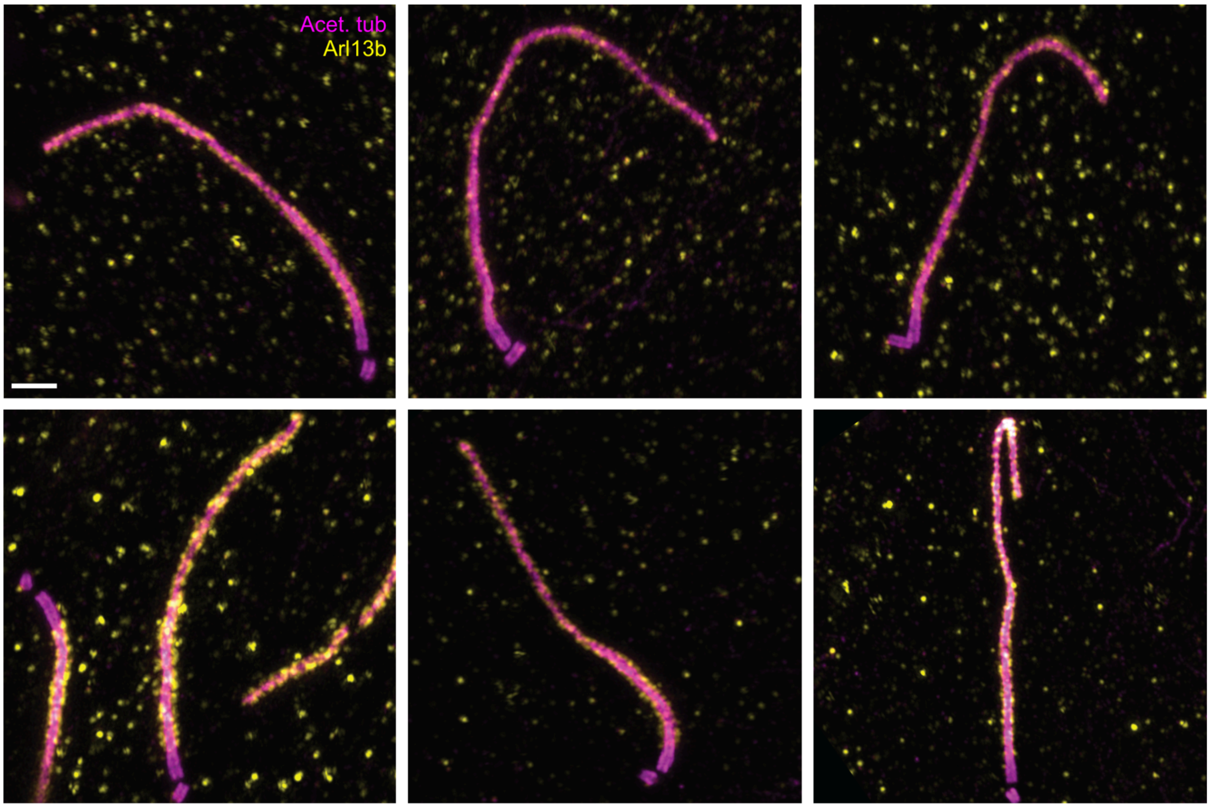

Supplementary Figure 4: Variability in islet cell primary cilia curvature in UExM images.
Scale bar: 1 µm.

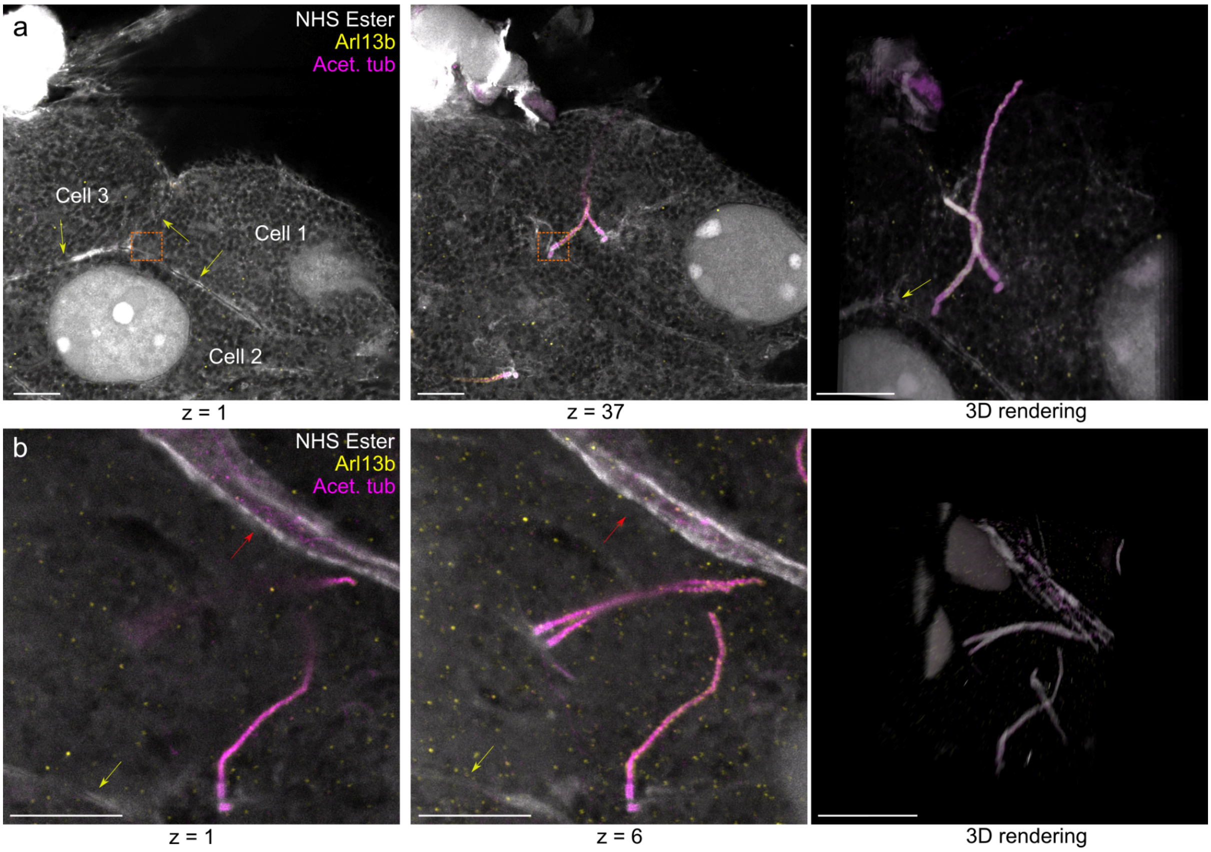

Supplemental Figure 5. Validating double ciliated cells by U-ExM.

a) Representative individual slices through a z-stack of a double ciliated cell, as determined by tracking the cell borders identified by NHS Ester (yellow arrows). The position of one of the basal bodies is indicated by the orange box. 3D rendering clearly identifies both basal bodies in the cytoplasm of the same cell. b) In some cases, oOnly one border can be differentiated by NHS Ester staining (yellow arrow), while the other border can be inferred by a juxtaposed blood vessel (red arrow). In these cases, double ciliation is inferred from the close proximity of the basal bodies. Acetylated tubulin (magenta), Arl13b (yellow) and NHS ester (grays). Scale bars: 2.5 µm.

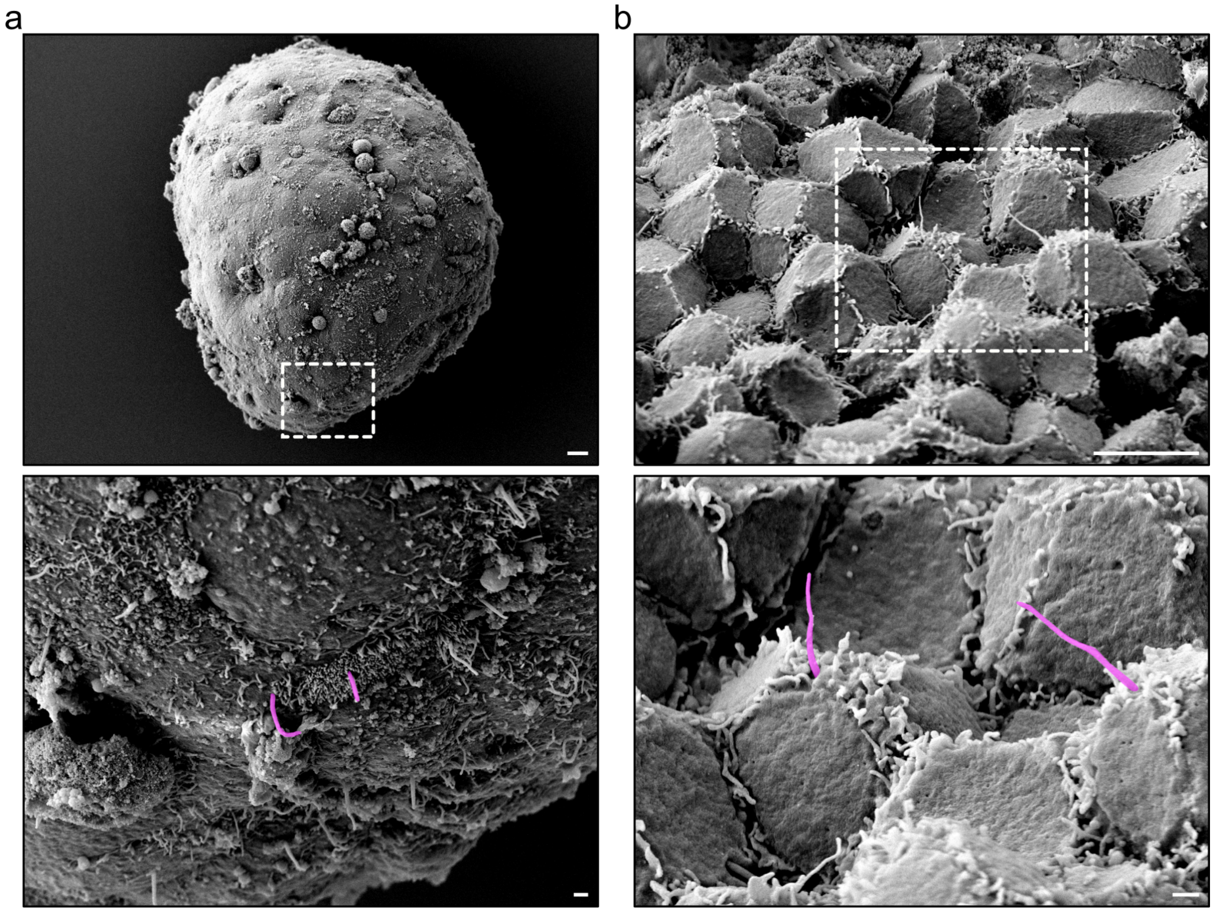

Supplementary Figure 6: SEM of isolated intact and broken mouse islets.
a) Intact islet with magnification of boxed area showing islet cell primary cilia (purple). b) Broken islet with visible edges and microvilli of the islet cells as well as primary cilia (purple). Scale bars: 1 µm.

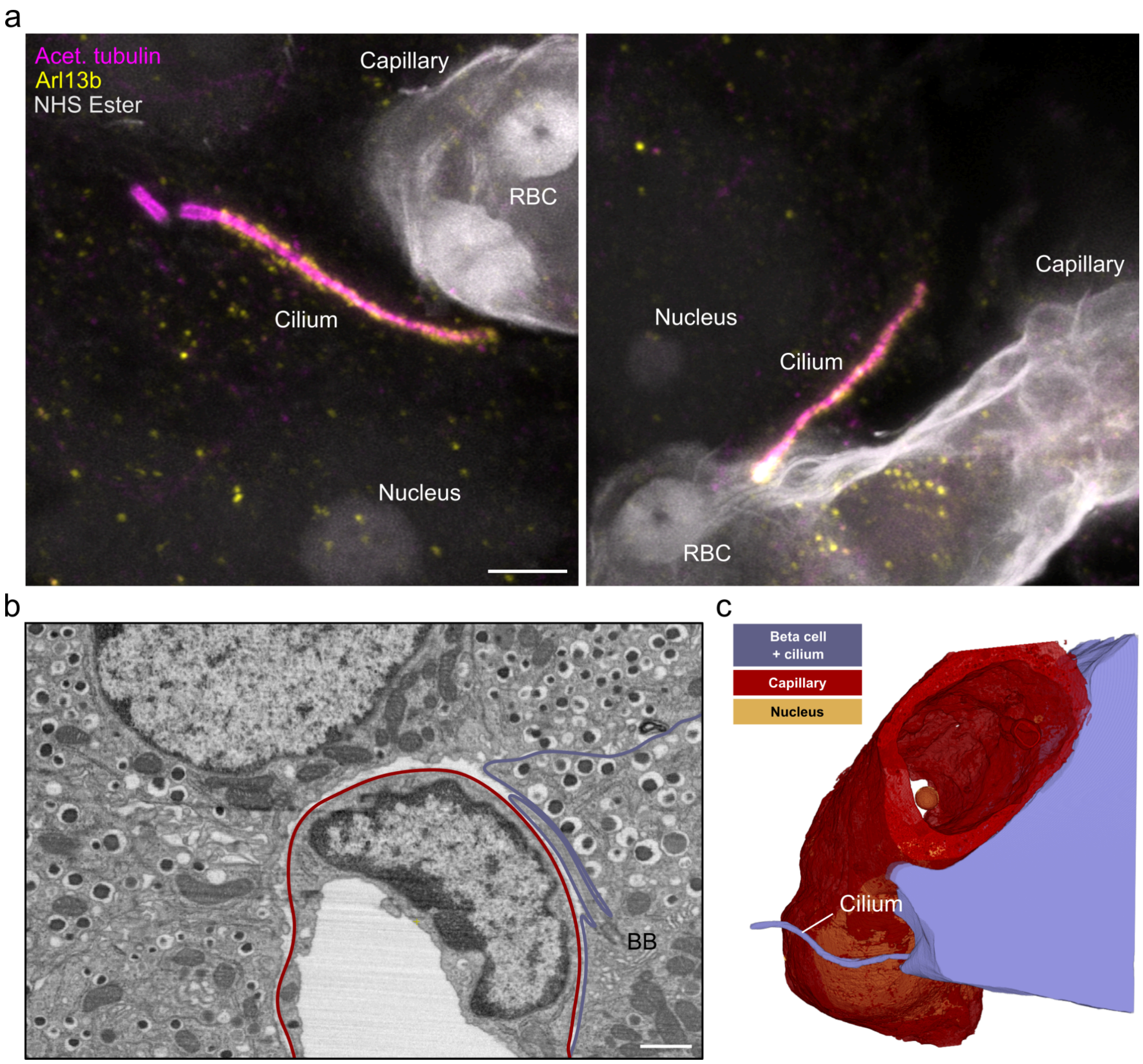

Supplementary Figure 7: Interaction of islet cell primary cilia with blood vessels.
a) Expanded beta cell cilia in close contact with blood vessels, as visualized by acetylated tubulin (magenta) Arl13b (yellow) and NHS Ester (gray). Scale bar: 1 µm. b) FIB-SEM slice showing a blood vessel with adjacent islet cells. A beta cell projects its primary cilium along the endothelial cells forming the vessel. Scale bar: 500 nm. c) The 3D rendering shows the primary cilium projecting along the vessel.

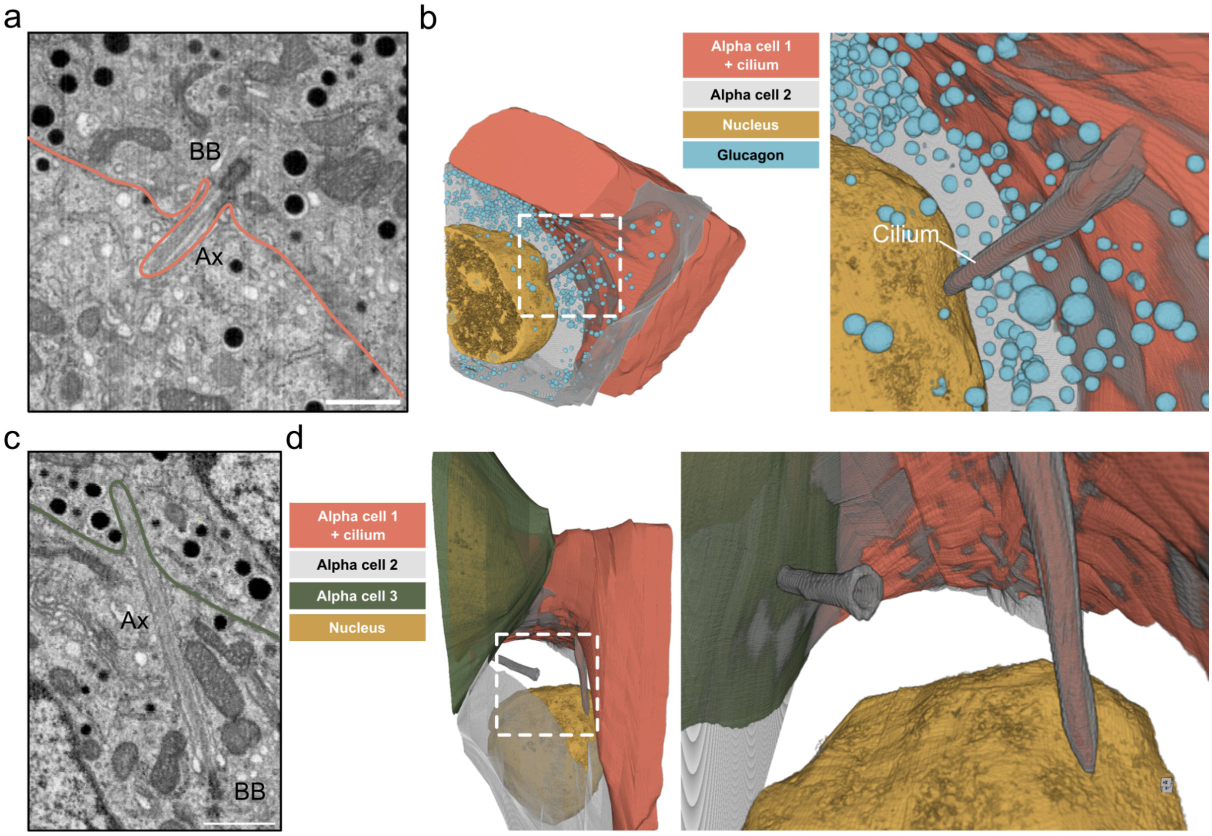

Supplementary Figure 8: Alpha cell primary cilium “pinches” a neighboring alpha cell.
a) FIB-SEM slice showing an alpha cell primary cilium with basal body (BB) and axoneme) pushing into a neighboring alpha cell. Scale bar: 500 nm b) 3D rendering of both cells with inset depicting the termination of the primary cilium close to the nucleus. c) A FIB-SEM slice through the gray alpha cell depicted in b) with its cilium pinching into the neighboring alpha cell (green). Scale bar: 500 nm. d) 3D renderings of all 3 alpha cells with 2 cilia protruding into the adjacent cell.

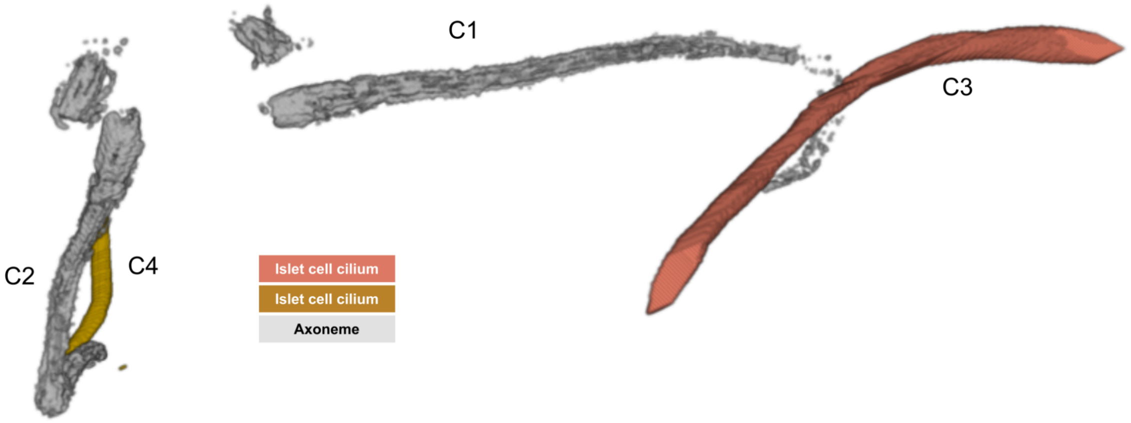

Supplementary Figure 9: Cilia in contact with neighboring cilia.
3D renderings of the two primary cilia (C1 and C2) of a double-ciliated beta cell which are in close proximity to cilia (C3 and C4) from neighboring islet cells on opposite sides of the beta cell. Only the tips of C1 and C2 reach the cell exterior space where they contact C3 and C4.

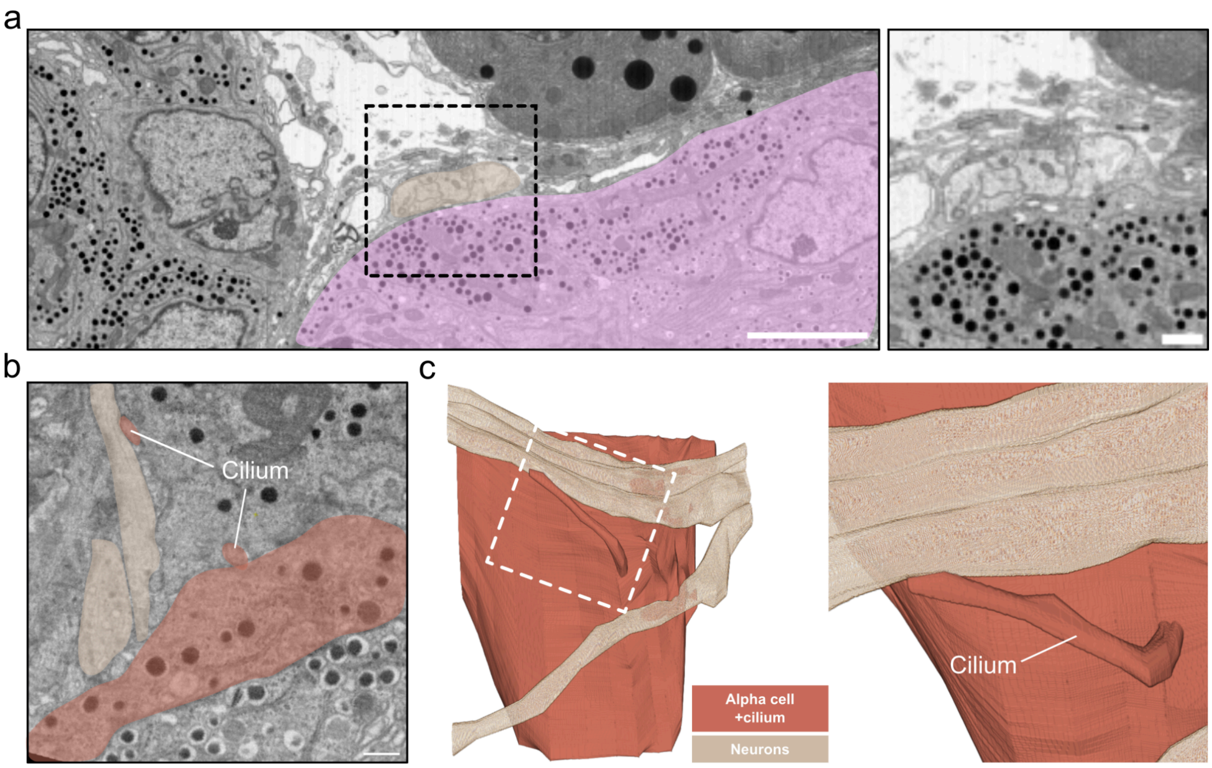

Supplementary Figure 10: Connection of alpha cell primary cilium tip with neurons.
a) Slices through a FIB-SEM volume showing axons (beige) connecting to a pancreatic islet (magenta). Scale bar: 5 µm. Next is the magnification of the boxed area showing the different neurons in contact with an alpha cell. Scale bar: 1 µm. b) Slice through a FIB-SEM volume with nerves (beige) and an alpha cell and its cilium (orange). Scale bar: 500 nm c) 3D renderings of nerves and the alpha cell. The magnified view shows the connections between the cilium tip and the neuron.

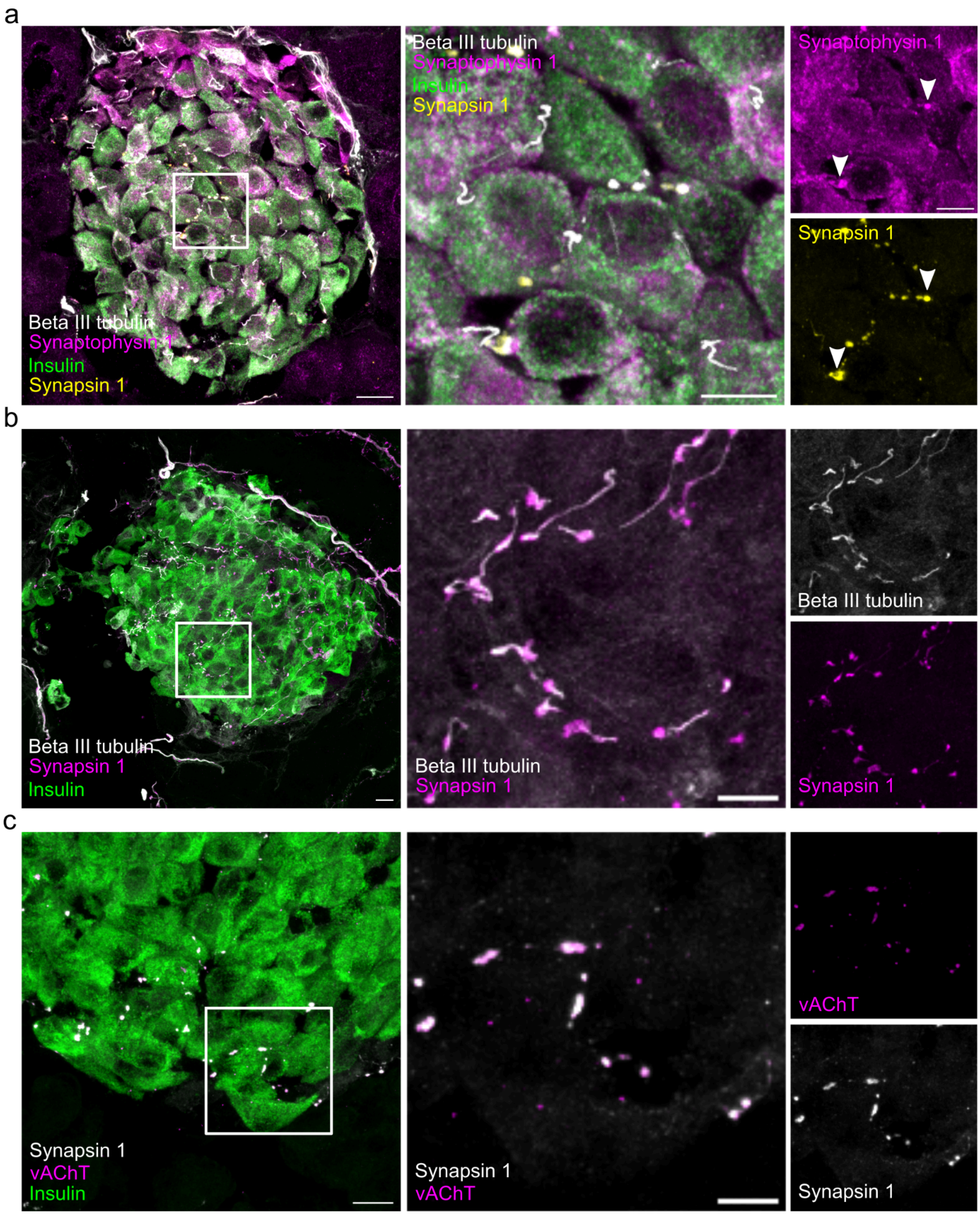

Supplementary Figure 11: Characterization of presynaptic markers on islet innervation.
a) Maximum intensity confocal images of a cryo section of mouse pancreas after immunofluorescence labeling for insulin (green), beta III tubulin (white), synaptophysin 1 (magenta), and synapsin 1 (yellow). Scale bar 10 µm. The center image shows the magnified marked region. Scale bar: 5 µm. The right images show single channels of synaptophysin 1 and synapsin 1 with arrowheads pointing to regions of colocalization. b) Maximum intensity confocal images of a cryo section of mouse pancreas after immunofluorescence labeling for insulin (green), beta III tubulin (white), and synapsin 1 (magenta). Scale bar 10 µm. The center image shows the magnified marked region without the insulin channel. Scale bar: 5 µm. The right images show single channels of beta III tubulin and synapsin 1. c) Maximum intensity confocal images of a cryo section of mouse pancreas after immunofluorescence labeling for insulin (green), synapsin 1 (white), and vAChT (magenta). Scale bar 10 µm.

The center image shows the magnified marked region without the insulin channel. Scale bar: 5 µm. The right images show single channels of vAChT and synapsin 1.

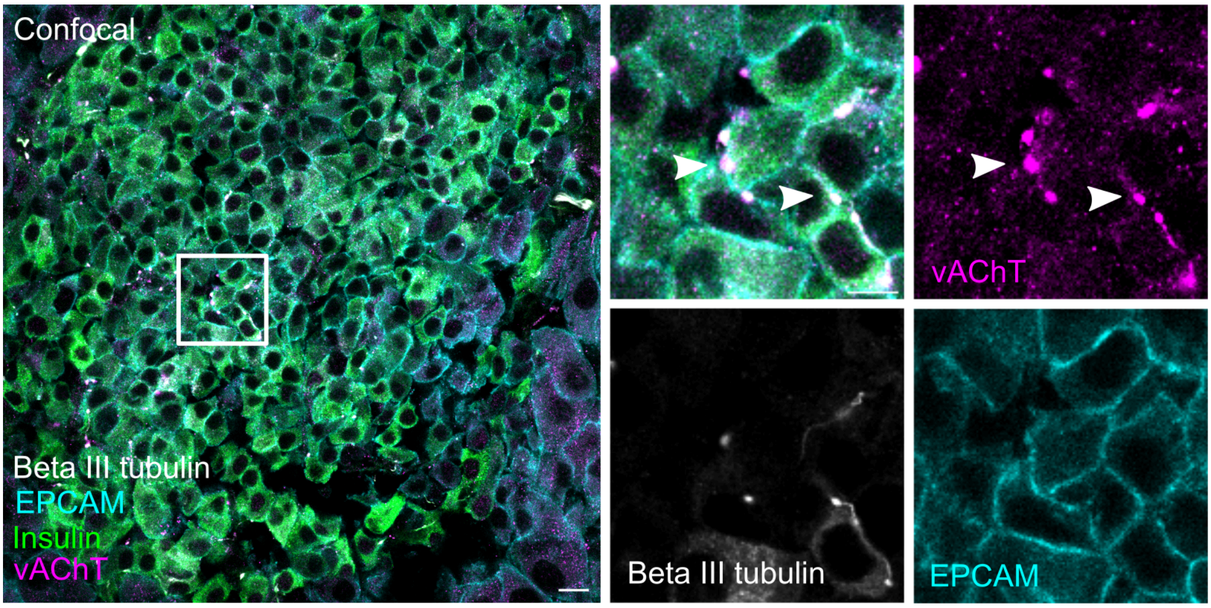

Supplementary Figure 12: Synaptic connections to the beta cell membrane.
Single confocal slice of a mouse pancreas section stained for insulin (green), beta III tubulin (white), vAChT (magenta), and EPCAM (cyan). Scale bar: 10 µm. Magnified images of the marked area show the localization of vAChT positive signal at the plasma membranes of beta cells. Scale bar: 5 µm.
