# Supplementary material for: Structure, interaction and nervous connectivity of beta cell primary cilia
Source: Nat Commun. 2024 Oct 24;15:9168. doi: 10.1038/s41467-024-53348-5 (PMC11502866; doi:10.1038/s41467-024-53348-5)
Supplement: Supplementary file 3 — Description of Additional Supplementary Files [file 41467_2024_53348_MOESM3_ESM.pdf]

<u>Description of Additional Supplementary Files</u>

Supplementary Movie 1: 3D rendering of mouse beta cell cilium within islet tissue.

Supplementary Movie 2: Raw ssET of a human beta cell primary cilium.

Supplementary Movie 3: FIB-SEM + Axoneme reconstruction of a mouse beta cell primary cilium.

Supplementary Movie 4: Spatial restriction of beta cell primary cilia.

Supplementary Movie 5: Beta cell axo-ciliary synapse.
